# Thermodynamics of Concomitant Charge‐Transfer, Antiferromagnetic, and Isostructural Phase Transitions in Barocaloric ACu_3_Fe_4_O_12_ Quadruple Perovskites

**DOI:** 10.1002/advs.202516375

**Published:** 2026-08-21

**Authors:** Jefferson Delgado‐Quesada, Gian Giacomo Guzmán‐Verri

**Affiliations:** ^1^ Centro de Investigación en Ciencia e Ingeniería de Materiales Universidad de Costa Rica San José Costa Rica; ^2^ Escuela de Física Universidad de Costa Rica San José Costa Rica; ^3^ Centro de Investigación en Matemática Pura y Aplicada Universidad de Costa Rica San José Costa Rica

**Keywords:** barocaloric effects, charge‐transfer transitions, landau theory, quadruple perovskites

## Abstract

We develop a minimal Landau theory of the concomitant intersite charge‐transfer, antiferromagnetic, and isostructural phase transitions in the barocaloric quadruple perovskites ${\mathrm{ACu}}_{3}{\mathrm{Fe}}_{4}O_{12}$ (A = La, Pr, Nd, Sm, Eu, Gd, Tb). A non‐symmetry‐breaking order parameter for the Cu–Fe intersite transfer is taken as primary, with the ${\mathrm{Fe}}^{3+}$ staggered magnetization and the volume strain enslaved to the charge transfer through magnetoelastic and charge‐strain couplings. The volume and entropy discontinuities, the thermal expansivities and bulk moduli of the two phases, and the slope of the pressure–temperature phase boundary are thereby tied to one another through the model couplings. The model yields a pressure‐independent transition entropy, so the latent heat is proportional to the transition temperature, accounting for its near‐constancy under A‐site substitution. Applied to ${\mathrm{NdCu}}_{3}{\mathrm{Fe}}_{4}O_{12}$, the theory reproduces the charge, magnetic, and structural responses across the transition from parameters fixed by experimental data, and resolves the transition entropy into comparable charge and magnetic contributions by anchoring the magnetic sector on the intrinsic ordering scale of the charge‐transferred state. The barocaloric effect is inverse, with a peak isothermal entropy change at 0.5 GPa of $≃73\;J\;K^{-1}\;{\mathrm{kg}}^{-1}$, comparable to the quasi‐direct value of $65\;J\;K^{-1}\;{\mathrm{kg}}^{-1}$. The theory makes explicit the intrinsic thermal expansion of each phase, a contribution omitted in the entropy construction underlying the quasi‐direct determination.

## Graphical abstract

A copper‐iron charge transfer instability drives concomitant electronic, magnetic, and isostructural phase transitions in ${\mathrm{ACu}}_{3}{\mathrm{Fe}}_{4}O_{12}$, with an inverse barocaloric effect. A minimal Landau theory ties these thermodynamic signatures together, revealing a pressure‐independent transition entropy with comparable charge and magnetic contributions.

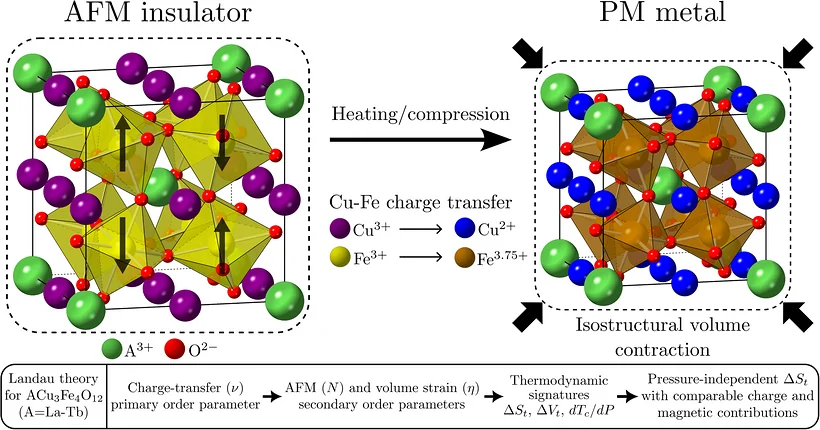

## 1 Introduction

Quadruple perovskites (QPs) are complex oxides with the chemical formula ${\mathrm{AA}}_{3}^{\prime }B_{4}O_{12}$, featuring ordered arrangements of multiple cations on the A and B sites of the perovskite lattice [1, 2]. Their unique crystal structures enable a wide range of physical and functional phenomena, including large magnetoresistance [3, 4], giant dielectricity [5, 6], magnetoelectricity [7], and catalysis [8].

**FIGURE 1:**
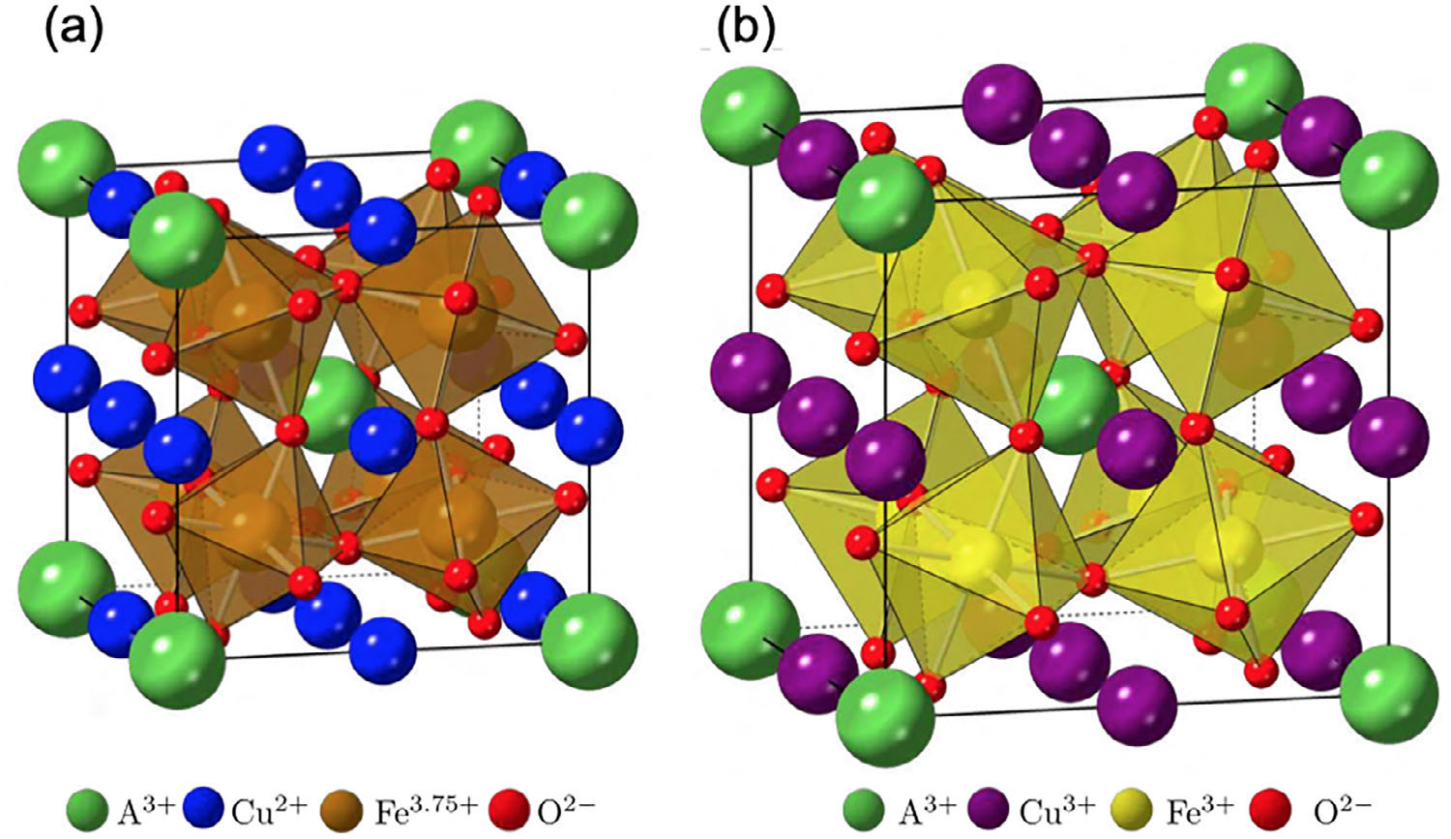
Crystal structure of ACFO in the (a) high‐temperature and (b) low‐temperature phases, both adopting $Im\bar{3}$ symmetry. The change in volume is not to scale and shown schematically for clarity. Generated using CrystalMaker.

In ${\mathrm{ACu}}_{3}{\mathrm{Fe}}_{4}O_{12}$ (ACFO) [9, 10, 11, 12], where the A‐site is occupied by a trivalent lanthanide (La, Pr, Nd, Sm, Eu, Gd, Tb), the high‐temperature (HT) phase crystallizes into a cubic $Im\bar{3}$ lattice structure with the nominal ionic formula $A^{3+}{\mathrm{Cu}}_{3}^{2+}{\mathrm{Fe}}_{4}^{3.75+}O_{12}$ (Figure 1a). Upon cooling, ACFO undergoes an intersite charge transfer (ICT) phase transition in which the $A^{\prime }$‐site ${\mathrm{Cu}}^{2+}$ oxidizes to ${\mathrm{Cu}}^{3+}$, releasing electrons that are transferred to the B‐site Fe cations, thereby reducing ${\mathrm{Fe}}^{3.75+}$ to ${\mathrm{Fe}}^{3+}$. The nominal valence state in the low‐temperature (LT) phase becomes $A^{3+}{\mathrm{Cu}}_{3}^{3+}{\mathrm{Fe}}_{4}^{3+}O_{12}$, and the $Im\bar{3}$ space group is retained after a ≃1‐2 % volume expansion on cooling at the phase transition (Figure 1b), characteristic of negative thermal expansion. The ICT is concomitant with first‐order metal‐to‐insulator and paramagnetic‐to‐antiferromagnetic transitions. The antiferromagnetic (AFM) ordering is G‐type, with each B‐site ${\mathrm{Fe}}^{3+}$ moment aligning antiparallel to its six nearest iron neighbors and the ordered moments lying along one of the cube axes. No magnetic contribution is observed from the A‐site trivalent lanthanide or the $A^{\prime }$‐site ${\mathrm{Cu}}^{3+}$.

The driving mechanism remains debated. First‐principles studies variously attribute the primary instability to lattice expansion of the Cu–O bonds [13], to a change in the Fe spin state [14], or to a simultaneous coupling of charge, spin, and lattice degrees of freedom [15, 16], while a Falicov–Kimball model [17] described the charge and spin order as a single combined instability (though no lattice effects were considered). Here, we adopt the empirical view [9, 18, 19] that the ICT is the primary driving instability, with the isostructural and AFM transitions arising through its coupling to the lattice and spin sectors. The ${\mathrm{Fe}}^{3.75+}$ sublattice of the HT phase is in a delocalized mixed‐valence state, which does not support a network of well‐defined local moments. When the ICT occurs, reduction of the B‐site Fe valence expands the Fe–O bonds (driving the isostructural volume change) [19, 20], and the resulting ${\mathrm{Fe}}^{3+}$ ions carry robust local moments (≃ 3–4 $\mu_{B}$ per ${\mathrm{Fe}}^{3+}$) [19, 21], that order in the G‐type pattern via Fe–O–Fe superexchange. The agreement between our theory and experiment demonstrated below supports this hypothesis.

Isovalent A‐site cation substitution across the lanthanide series provides control over the transition temperature $T_{c}$ in ACFO, which varies from ≃360 K to ≃240 K as the lanthanide size decreases [10]. The transition entropies are large (≃60‐84 $J\;K^{-1}\;{\mathrm{kg}}^{-1}$), making QPs attractive for solid‐state thermal energy storage and thermal energy conversion [21, 22]. Indeed, nominally reversible [23] thermal changes driven by hydrostatic pressure [24, 25, 26] have been quasi‐directly determined in ${\mathrm{NdCu}}_{3}{\mathrm{Fe}}_{4}O_{12}$ (NCFO, $T_{c}≃310\;K$) [21] and in related compounds [27, 28]. Such barocaloric (BC) effects [29, 30, 31] are parametrized by isothermal changes in entropy and adiabatic changes in temperature, which for NCFO peak at ≃65 $JK^{-1}{\mathrm{kg}}^{-1}$ and ≃ 14 K under 0.5 GPa at ≃294 K. These values are comparable to the largest changes in inorganic solid materials [32].

Here, we develop a minimal Landau theory that captures the temperature and pressure dependence of the thermodynamic features near the ICT transition across the ACFO family. We take a non‐symmetry‐breaking charge‐transfer order parameter as primary and enslave the staggered magnetization and the volume strain to it through magnetoelastic and charge‐strain couplings; eliminating these secondary order parameters leaves an effective free energy whose minimum fixes the thermodynamics. A physically motivated ansatz, justified by the abrupt charge transfer observed across the family, then reduces the relative volume, entropy, staggered magnetization, and susceptibility to closed forms in terms of measurable quantities, and permits a direct inversion of the experimental data. We apply the framework to NCFO, calculate the associated barocaloric effects, and compare throughout with available measurements. The same model exposes its own limits: we trace a discrepancy in the ordering of the two bulk moduli to the linear strain coupling, and identify experiments that would test the theory.

## 2 Model

### 2.1 Order Parameters

We introduce a non‐symmetry‐breaking charge‐transfer primary order parameter (OP) ν that parameterizes the formal charge redistribution as

$$\begin{array}{c} A^{3+}{\mathrm{Cu}}_{3}^{2+\nu }{\mathrm{Fe}}_{4}^{3.75-\frac{3}{4}\nu }O_{12}, \end{array}$$

with ν≃0 in the high‐temperature phase and ν≃1 in the low‐temperature phase; the coefficient 3/4 enforces charge balance across the three Cu and four Fe sites. For the magnetic sector we introduce a staggered (Néel) magnetization N, describing the G‐type AFM order of the B‐site ${\mathrm{Fe}}^{3+}$ moments, and a uniform magnetization M, included as an auxiliary OP to access the ferromagnetic (FM) susceptibility. We also include the elastic volume strain η, which captures lattice deformations arising from the ICT, AFM ordering, thermal expansion, and applied hydrostatic pressure.

We treat ν as the primary OP and N, M, and η as secondary, eliminating them in two stages. We first eliminate η at fixed N, M, and ν: being associated with long‐wavelength acoustic phonons, whose frequencies vanish in the long‐wavelength limit [33], η relaxes to its equilibrium value for any given charge and magnetic configuration. We then eliminate N and M at fixed ν. This second step reflects the physical picture adopted here: the ${\mathrm{Fe}}^{3+}$ local moments are created by the ICT (the mixed‐valence ${\mathrm{Fe}}^{3.75+}$ sublattice of the HT phase carries no well‐defined local moments), so the magnetic order is enslaved to the charge‐transfer OP rather than independently driven. The result is an effective free energy $\hat{G}\left(\nu \right)$ governing the primary phase transition.

### 2.2 Free Energy Density

We consider an isotropic solid subjected to an applied hydrostatic pressure P, where P=0 corresponds to ambient pressure. The Landau free energy density G can include both even and odd terms in ν, as the intersite charge‐transfer OP is not associated with a symmetry operation that maps ν onto −ν. In addition, G must be invariant under time reversal and under exchange of the two equivalent Fe sublattices. Since (N,M) transforms as (N,M)→(−N,−M) under time reversal and as (N,M)→(−N,M) under sublattice exchange, the free energy is even separately in N and M. Based on these symmetry constraints, we decompose the free‐energy density into electronic, magnetic, elastic, and coupling contributions as follows,

(1) $$\begin{array}{c} G=G_{0}+G_{\mathrm{charge}}+G_{\mathrm{magnetic}}+G_{\mathrm{strain}}\\ +G_{\text{charge-strain}}+G_{\text{charge-magnetic}}+G_{\text{magnetic-strain}}+P\;\eta , \end{array}$$

where $G_{0}$ is the free‐energy density of the background, $G_{\mathrm{charge}}$ is the ICT free‐energy density [34],

$$\begin{array}{c} G_{\mathrm{charge}}=a_{1}\left(T\right)\nu +\frac{a_{2}}{2}\nu^{2}+\frac{a_{3}}{3}\nu^{3}+\frac{a_{4}}{4}\nu^{4}+\frac{a_{5}}{5}\nu^{5}+\frac{a_{6}}{6}\nu^{6}, \end{array}$$

$G_{\mathrm{magnetic}}$ is the magnetic energy,

$$\begin{array}{c} G_{\mathrm{magnetic}}=\frac{b_{2}\left(T\right)}{2}N^{2}+\frac{b_{2}\left(T\right)+\lambda }{2}M^{2}+\frac{b_{4}}{4}\left(N^{4}+M^{4}+6N^{2}M^{2}\right), \end{array}$$

$G_{\mathrm{strain}}$ is the elastic energy, which includes the thermal expansion,

$$\begin{array}{c} G_{\mathrm{strain}}=-B\alpha \left(T-T_{0}^{\prime }\right)\eta +\frac{B}{2}\eta^{2}, \end{array}$$

$G_{\text{charge-strain}}$ is the charge‐strain coupling energy,

$$\begin{array}{c} G_{\text{charge-strain}}=g_{2}\eta \nu , \end{array}$$

$G_{\text{charge-magnetic}}$ is the charge‐magnetic coupling energy,

$$\begin{array}{c} G_{\text{charge-magnetic}}=-\frac{\nu }{2}\left(g_{1N}N^{2}+g_{1M}M^{2}\right), \end{array}$$

and $G_{\text{magnetic-strain}}$ is the magnetostrictive coupling energy,

$$\begin{array}{c} G_{\text{magnetic-strain}}=\frac{\eta }{2}\left(g_{3N}N^{2}+g_{3M}M^{2}\right). \end{array}$$

Here, $a_{1}=a_{10}\left(T-T_{\nu }\right)$ and $b_{2}=b_{20}\left(T-T_{N}\right)$; the same bare quadratic coefficient $b_{2}\left(T\right)$ governs both N and M, reflecting their common origin in the Fe moments, with the FM channel offset by λ. The quantities $a_{10},b_{20},T_{\nu },T_{N},a_{2},a_{3},a_{4},a_{5},a_{6},b_{4},g_{1N},g_{1M},g_{2},g_{3N},g_{3M}$, and λ are model parameters independent of temperature and pressure; α is the bare coefficient of volumetric expansion, and B is the bare bulk modulus. We require $a_{6}>0$ and $b_{4}>0$ for G to be bounded from below. $T_{0}^{\prime }$ is a reference temperature at which the undeformed state (η=0) is defined [35]; we take this undeformed reference state to be at $T_{0}^{\prime }$ and ambient pressure.

At thermodynamic equilibrium, the solid relaxes to a stress‐free state such that ${\left(\partial G/\partial \eta \right)}_{N,M,\nu }=0$ [36], which gives

(2) $$\begin{array}{c} \eta =\alpha \left(T-T_{0}^{\prime }\right)-\frac{P}{B}-\frac{g_{2}}{B}\nu -\frac{1}{2B}\left(g_{3N}N^{2}+g_{3M}M^{2}\right). \end{array}$$

Substitution of Equation (2) into Equation (1) gives the effective free‐energy density,

(3) $$\begin{array}{c} \tilde{G}=G_{0}+{\tilde{G}}_{\mathrm{charge}}+{\tilde{G}}_{\mathrm{magnetic}}+{\tilde{G}}_{\text{charge-magnetic}}-\frac{{\left[P-B\alpha \left(T-T_{0}^{\prime }\right)\right]}^{2}}{2B}, \end{array}$$

where

$$\begin{array}{cc} {\tilde{G}}_{\mathrm{charge}} & ={\tilde{a}}_{1}\left(T,P\right)\nu +\frac{{\tilde{a}}_{2}}{2}\nu^{2}+\frac{a_{3}}{3}\nu^{3}+\frac{a_{4}}{4}\nu^{4}+\frac{a_{5}}{5}\nu^{5}+\frac{a_{6}}{6}\nu^{6},\\ {\tilde{G}}_{\mathrm{magnetic}} & =\frac{{\tilde{b}}_{2N}\left(T,P\right)}{2}N^{2}+\frac{{\tilde{b}}_{2M}\left(T,P\right)+\lambda }{2}M^{2}\\ & \;+\frac{1}{4}\left({\tilde{b}}_{4N}N^{4}+{\tilde{b}}_{4M}M^{4}+6{\tilde{b}}_{4NM}N^{2}M^{2}\right), \end{array}$$

and

$$\begin{array}{c} {\tilde{G}}_{\text{charge-magnetic}}=-\frac{\nu }{2}\left({\tilde{g}}_{1N}N^{2}+{\tilde{g}}_{1M}M^{2}\right), \end{array}$$

with renormalized parameters

(4a) $$\begin{array}{cc} {\tilde{a}}_{1}\left(T,P\right) & ={\tilde{a}}_{10}\left(T-{\tilde{T}}_{\nu }\right)-\frac{g_{2}}{B}P, \end{array}$$

(4b) $$\begin{array}{cc} {\tilde{a}}_{10} & =a_{10}+g_{2}\alpha , \end{array}$$

(4c) $$\begin{array}{cc} {\tilde{T}}_{\nu } & =T_{\nu }+\frac{g_{2}\alpha }{{\tilde{a}}_{10}}\left(T_{\nu }-T_{0}^{\prime }\right), \end{array}$$

(4d) $$\begin{array}{cc} {\tilde{a}}_{2} & =a_{2}-\frac{g_{2}^{2}}{B}, \end{array}$$

(4e) $$\begin{array}{cc} {\tilde{b}}_{2N}\left(T,P\right) & ={\tilde{b}}_{20N}\left(T-{\tilde{T}}_{N}\right)-\frac{g_{3N}}{B}P, \end{array}$$

(4f) $$\begin{array}{cc} {\tilde{b}}_{2M}\left(T,P\right) & ={\tilde{b}}_{20M}\left(T-{\tilde{T}}_{M}\right)-\frac{g_{3M}}{B}P, \end{array}$$

(4g) $$\begin{array}{cc} {\tilde{b}}_{20N} & =b_{20}+g_{3N}\alpha , \end{array}$$

(4h) $$\begin{array}{cc} {\tilde{b}}_{20M} & =b_{20}+g_{3M}\alpha , \end{array}$$

(4i) $$\begin{array}{cc} {\tilde{T}}_{N} & =T_{N}-\frac{g_{3N}\alpha }{{\tilde{b}}_{20N}}\left(T_{N}-T_{0}^{\prime }\right), \end{array}$$

(4j) $$\begin{array}{cc} {\tilde{T}}_{M} & =T_{N}-\frac{g_{3M}\alpha }{{\tilde{b}}_{20M}}\left(T_{N}-T_{0}^{\prime }\right), \end{array}$$

(4k) $$\begin{array}{cc} {\tilde{b}}_{4N} & =b_{4}-\frac{g_{3N}^{2}}{2B}, \end{array}$$

(4l) $$\begin{array}{cc} {\tilde{b}}_{4M} & =b_{4}-\frac{g_{3M}^{2}}{2B}, \end{array}$$

(4m) $$\begin{array}{cc} {\tilde{b}}_{4NM} & =\frac{{\tilde{b}}_{4N}+{\tilde{b}}_{4M}}{2}+\frac{1}{4B}\left(g_{3N}^{2}+g_{3M}^{2}-\frac{2}{3}g_{3N}g_{3M}\right), \end{array}$$

(4n) $$\begin{array}{cc} {\tilde{g}}_{1N} & =g_{1N}+\frac{g_{2}g_{3N}}{2B}, \end{array}$$

(4o) $$\begin{array}{cc} {\tilde{g}}_{1M} & =g_{1M}+\frac{g_{2}g_{3M}}{2B}. \end{array}$$

We note that the renormalized parameters ${\tilde{a}}_{1}\left(T,P\right)$, ${\tilde{b}}_{2N}\left(T,P\right)$, and ${\tilde{b}}_{2M}\left(T,P\right)$ depend on pressure as a consequence of the charge–strain and magnetoelastic couplings. Although N and M share the same bare quadratic coefficient, the magnetostrictive couplings $g_{3N}\neq g_{3M}$ renormalize them differently, so that the AFM and FM channels acquire distinct effective stiffnesses ${\tilde{b}}_{2N}$ and ${\tilde{b}}_{2M}$ and distinct characteristic temperatures ${\tilde{T}}_{N}$ and ${\tilde{T}}_{M}$.

For fixed ν, the equilibrium values N(T,P) and M(T,P) are given by ${\left(\partial \tilde{G}/\partial N\right)}_{M,\nu }=0$ and ${\left(\partial \tilde{G}/\partial M\right)}_{N,\nu }=0$, which in the absence of FM order yield

(5) $$\begin{array}{c} M\left(T,P\right)=0,\;N\left(T,P\right)=\left\{\begin{array}{cc} 0, & r_{N}\geq 0,\\ \pm \sqrt{-\;r_{N}\left(\nu ,T,P\right)/{\tilde{b}}_{4N}}, & r_{N}\leq 0, \end{array}\right. \end{array}$$

where $r_{N}\left(\nu ,T,P\right)\equiv {\tilde{b}}_{2N}\left(T,P\right)-{\tilde{g}}_{1N}\;\nu$ is the renormalized magnetic stiffness, and the sign of N is immaterial, $\tilde{G}$ being even in N. Equation (5) shows that AFM order requires the equilibrium value of ν to satisfy $r_{N}\left(\nu ,T,P\right)<0$, i.e., a sufficiently large equilibrium ν drives $r_{N}$ negative through the ICT coupling ${\tilde{g}}_{1N}\nu$, triggering the AFM order. Otherwise, the paramagnetic (PM) phase prevails.

Upon substituting Equation (5) into Equation (3), the effective free energy splits into two branches: a PM branch ($r_{N}\geq 0$) in which the magnetic sector reduces to zero, and an AFM branch ($r_{N}\leq 0$) in which the magnetic sector collapses to $-r_{N}^{2}/\left(4{\tilde{b}}_{4N}\right)$. Expanding $r_{N}^{2}={\left({\tilde{b}}_{2N}-{\tilde{g}}_{1N}\nu \right)}^{2}$ renormalizes the coefficients of $\nu^{0}$, $\nu^{1}$, and $\nu^{2}$, so that

(6) $$\begin{array}{ccc} \hat{G} & = & G_{0}+{\hat{a}}_{1}\left(T,P\right)\;\nu +\frac{{\hat{a}}_{2}\left(T,P\right)}{2}\nu^{2}+\frac{a_{3}}{3}\nu^{3}+\frac{a_{4}}{4}\nu^{4}+\frac{a_{5}}{5}\nu^{5}\\ & & +\;\frac{a_{6}}{6}\nu^{6}+\hat{\delta }\left(T,P\right)-\frac{{\left[P-B\alpha \left(T-T_{0}^{\prime }\right)\right]}^{2}}{2B}, \end{array}$$

with

(7) $${\hat{a}}_{1}={\tilde{a}}_{1}+\frac{{\tilde{g}}_{1N}\;{\tilde{b}}_{2N}}{2{\tilde{b}}_{4N}},\;{\hat{a}}_{2}={\tilde{a}}_{2}-\frac{{\tilde{g}}_{1N}^{2}}{2{\tilde{b}}_{4N}},\;\hat{\delta }=-\frac{{\tilde{b}}_{2N}^{2}}{4{\tilde{b}}_{4N}},$$

for $r_{N}\leq 0$, and ${\hat{a}}_{1}={\tilde{a}}_{1}$, ${\hat{a}}_{2}={\tilde{a}}_{2}$, $\hat{\delta }=0$ for $r_{N}\geq 0$.

The equilibrium value ν(T,P) is given by the stationarity condition $\partial \hat{G}/\partial \nu =0$,

(8) $$\begin{array}{c} {\hat{a}}_{1}\left(T,P\right)+{\hat{a}}_{2}\left(T,P\right)\;\nu +a_{3}\nu^{2}+a_{4}\nu^{3}+a_{5}\nu^{4}+a_{6}\nu^{5}=0. \end{array}$$

We now turn to the thermodynamic properties. The relative volume $V={\left(\partial \hat{G}/\partial P\right)}_{T}$ is

(9) $$\begin{array}{c} V\left(T,P\right)=V_{0}+\alpha \left(T-T_{0}^{\prime }\right)-\frac{P}{B}-\frac{g_{2}}{B}\;\nu \left(T,P\right)-\frac{g_{3N}}{2B}\;N^{2}\left(T,P\right), \end{array}$$

where $V_{0}={\left(\partial G_{0}/\partial P\right)}_{T}$ is the background contribution. The entropy density $S=-{\left(\partial \hat{G}/\partial T\right)}_{P}$ is

(10) $$\begin{array}{c} S\left(T,P\right)=S_{0}+\alpha^{2}B\left(T-T_{0}^{\prime }\right)-\alpha P-{\tilde{a}}_{10}\;\nu \left(T,P\right)-\frac{{\tilde{b}}_{20N}}{2}\;N^{2}\left(T,P\right), \end{array}$$

where $S_{0}=-{\left(\partial G_{0}/\partial T\right)}_{P}$ is the background entropy density. The FM volume susceptibility χ(T,P) is given by $\chi^{-1}=\mu_{0}^{-1}{\left(\partial^{2}\tilde{G}/\partial M^{2}\right)}_{M=0}$,

(11) $$\begin{array}{c} \mu_{0}\;\chi^{-1}\left(T,P\right)={\tilde{b}}_{2M}\left(T,P\right)+\lambda -{\tilde{g}}_{1M}\;\nu \left(T,P\right)+3{\tilde{b}}_{4NM}\;N^{2}\left(T,P\right). \end{array}$$

In Equations (9)–(11), the terms proportional to ν(T,P) represent the charge contribution and those proportional to $N^{2}\left(T,P\right)$ represent the AFM contribution. The second term of Equation (9) is the bare thermal expansion and the third the bare compression; the second and third terms of Eq. (10) are the corresponding thermal‐expansion contribution to the entropy.

From Equation (10), we calculate the isothermal entropy change upon compression, ΔS(T,0→P) [30], and decompose it into charge, AFM, and bare thermal‐expansion contributions:

(12) $$\begin{array}{cc} \Delta S(T,0\to P) & =S(T,P)-S(T,0)\\ & =\Delta S_{\text{charge}}+\Delta S_{\text{AFM}}+\Delta S_{\text{thermal exp.}}, \end{array}$$

where $\Delta S_{\text{charge}}={\tilde{a}}_{10}\;\left[\nu \left(T,0\right)-\nu \left(T,P\right)\right]$, $\Delta S_{\text{AFM}}=\left({\tilde{b}}_{20N}/2\right)\;\left[N^{2}\left(T,0\right)-N^{2}\left(T,P\right)\right]$, and $\Delta S_{\text{thermal exp.}}=-\alpha P$.

### 2.3 Phase Transition

#### 2.3.1 Phase Boundary

We determine the phase boundary $T_{c}\left(P\right)$ from the coexistence and stationarity conditions at the first‐order transition. Let $\nu_{H}\equiv \nu \left(T_{c}^{+},P\right)$ and $\nu_{L}\equiv \nu \left(T_{c}^{-},P\right)$ denote the charge‐transfer order‐parameter values in the HT and LT phases at the transition. At $T_{c}$, the coexistence condition $\hat{G}\left(\nu_{H},T_{c},P\right)=\hat{G}\left(\nu_{L},T_{c},P\right)$ yields

(13) $$\begin{array}{ccc} & & {\tilde{a}}_{1c}\;\nu_{H}+\frac{{\tilde{a}}_{2}}{2}\nu_{H}^{2}+\frac{a_{3}}{3}\nu_{H}^{3}+\frac{a_{4}}{4}\nu_{H}^{4}+\frac{a_{5}}{5}\nu_{H}^{5}+\frac{a_{6}}{6}\nu_{H}^{6}\\ & & \;=\left({\tilde{a}}_{1c}+\frac{{\tilde{g}}_{1N}\;{\tilde{b}}_{2Nc}}{2{\tilde{b}}_{4N}}\right)\nu_{L}+\frac{1}{2}\left({\tilde{a}}_{2}-\frac{{\tilde{g}}_{1N}^{2}}{2{\tilde{b}}_{4N}}\right)\nu_{L}^{2}\\ & & \;+\;\frac{a_{3}}{3}\nu_{L}^{3}+\frac{a_{4}}{4}\nu_{L}^{4}+\frac{a_{5}}{5}\nu_{L}^{5}+\frac{a_{6}}{6}\nu_{L}^{6}-\frac{{\tilde{b}}_{2Nc}^{2}}{4{\tilde{b}}_{4N}}, \end{array}$$

while the stationarity condition in Equation (8) gives

(14a) $$\begin{array}{cc} {\tilde{a}}_{1c}+{\tilde{a}}_{2}\nu_{H}+a_{3}\nu_{H}^{2}+a_{4}\nu_{H}^{3}+a_{5}\nu_{H}^{4}+a_{6}\nu_{H}^{5} & =0, \end{array}$$

(14b) $$\begin{array}{cc} & \left({\tilde{a}}_{1c}+\frac{{\tilde{g}}_{1N}\;{\tilde{b}}_{2Nc}}{2{\tilde{b}}_{4N}}\right)+\left({\tilde{a}}_{2}-\frac{{\tilde{g}}_{1N}^{2}}{2{\tilde{b}}_{4N}}\right)\nu_{L}+a_{3}\nu_{L}^{2}\\ & \;+\;a_{4}\nu_{L}^{3}+a_{5}\nu_{L}^{4}+a_{6}\nu_{L}^{5}=0, \end{array}$$

with ${\tilde{a}}_{1c}\equiv {\tilde{a}}_{1}\left(T_{c},P\right)$ and ${\tilde{b}}_{2Nc}\equiv {\tilde{b}}_{2N}\left(T_{c},P\right)$.

Empirically, the transition values $\nu_{H}$ and $\nu_{L}$ are independent of pressure [37], i.e., along the phase boundary $T=T_{c}\left(P\right)$,

$$\begin{array}{c} \frac{d\nu_{H}}{dP}=0,\;\frac{d\nu_{L}}{dP}=0. \end{array}$$

Taking the total derivative of the HT stationarity condition in Equation (14a) along the boundary, the term proportional to $d\nu_{H}/dP$ vanishes by the pressure‐independence of $\nu_{H}$, leaving $d{\tilde{a}}_{1c}/dP=0$; with Equation (4a) this fixes the slope as

(15) $$\begin{array}{cc} \frac{dT_{c}}{dP} & =\frac{1}{B}\frac{g_{2}}{{\tilde{a}}_{10}}. \end{array}$$

Differentiating the LT stationarity condition given in Equation (14b) along the boundary, the term proportional to $d\nu_{L}/dP$ likewise vanishes by the pressure‐independence of $\nu_{L}$, and the term $d{\tilde{a}}_{1c}/dP$ vanishes as above; since ${\tilde{g}}_{1N}\neq 0$, the remaining term requires $d{\tilde{b}}_{2Nc}/dP=0$, so that with Equation (4e)

(16) $$\begin{array}{cc} \frac{dT_{c}}{dP} & =\frac{1}{B}\frac{g_{3N}}{{\tilde{b}}_{20N}}. \end{array}$$

Equations (15) and (16) are two expressions for the single measured slope of the phase boundary [21] and must therefore coincide, which requires

(17) $$\begin{array}{c} \frac{g_{2}}{{\tilde{a}}_{10}}=\frac{g_{3N}}{{\tilde{b}}_{20N}}. \end{array}$$

This is the parameter constraint under which the ICT and AFM instabilities shift together under pressure and thus remain concomitant. Because $g_{2}$, ${\tilde{a}}_{10}$, $g_{3N}$, and ${\tilde{b}}_{20N}$ are independent of temperature and pressure, the slope $dT_{c}/dP$ is constant, and the transition temperature is linear in pressure,

(18) $$\begin{array}{c} T_{c}=T_{c0}+\frac{dT_{c}}{dP}P, \end{array}$$

where $T_{c0}$ is the transition temperature at ambient pressure.

Evaluated at P=0, Equations (13) and (14) fix $T_{c0}$ together with the remaining model parameters and enforce coincidence of the ICT and AFM transitions at ambient pressure; the equal slopes imposed by Equation (17) then ensure the two remain concomitant at finite P.

#### 2.3.2 Discontinuities

From Equations (5), (9), and (10), we calculate the discontinuities in the relative volume and entropy at the phase transition,

(19a) $$\begin{array}{cc} \Delta V_{t} & =V\left(T_{c}^{+},P\right)-V\left(T_{c}^{-},P\right)=-\frac{g_{2}}{B}\;\Delta \nu_{t}+\frac{g_{3N}}{2B}\;\Delta N_{t}^{2}, \end{array}$$

(19b) $$\begin{array}{cc} \Delta S_{t} & =S\left(T_{c}^{+},P\right)-S\left(T_{c}^{-},P\right)=-{\tilde{a}}_{10}\;\Delta \nu_{t}+\frac{{\tilde{b}}_{20N}}{2}\Delta N_{t}^{2}, \end{array}$$

where $\Delta \nu_{t}=\nu_{H}-\nu_{L}$ is the charge transfer at the transition and $\Delta N_{t}=N\left(T_{c}^{-},P\right)$ is the spontaneous Néel magnetization, with $\Delta N_{t}^{2}=-\left({\tilde{b}}_{2Nc}-{\tilde{g}}_{1N}\nu_{L}\right)/{\tilde{b}}_{4N}$ from Equation (5). The pressure‐independence of $\nu_{H}$, $\nu_{L}$, and ${\tilde{b}}_{2Nc}$ established above therefore renders $\Delta \nu_{t}$, $\Delta N_{t}$, and hence $\Delta S_{t}$ and $\Delta V_{t}$ pressure‐independent as well. The latent heat of the transition is $L=T_{c}\;\Delta S_{t}$; with $\Delta S_{t}$ pressure‐independent and $T_{c}$ linear in P (Equation (18)), L is linear in pressure.

Dividing Equation (19a) by Equation (19b) and imposing the concomitance constraint (17), $g_{3N}=g_{2}{\tilde{b}}_{20N}/{\tilde{a}}_{10}$, the terms in $\Delta \nu_{t}$ and $\Delta N_{t}^{2}$ cancel in the ratio, leaving $\Delta V_{t}/\Delta S_{t}=g_{2}/\left(B{\tilde{a}}_{10}\right)$. With Equation (15) this recovers the Clausius–Clapeyron equation,

(20) $$\begin{array}{c} \frac{dT_{c}}{dP}=\frac{\Delta V_{t}}{\Delta S_{t}}. \end{array}$$

Its recovery here is a consistency check: the phase‐boundary slope obtained from the stationarity conditions (Equations (15) and (16)) coincides with the thermodynamic ratio of the volume and entropy jumps, as it must.

### 2.4 Thermodynamic Properties Under a Step Ansatz

Experimentally, the intersite charge transfer in ACFO is essentially complete and abrupt: the Cu and Fe valences switch at $T_{c}$ and show no measurable temperature or pressure dependence away from the phase transition [9, 10, 37]. Guided by this observation, we take the charge transfer to occur entirely at the phase transition i.e.,

(21) $$\nu \left(T,P\right)≃\left\{\begin{array}{cc} 0, & T\geq T_{c},\\ 1, & T\leq T_{c}, \end{array}\right.$$

where $T_{c}$ is the pressure‐dependent transition temperature of Equation (18). Under Equation (21), $\nu_{H}=0$ and $\nu_{L}=1$, and Equations (5) and (9)–(12) take closed forms in terms of the model parameters, which allows the experimental data to be directly inverted (Section 2.5). The ansatz in Equation (21) is verified a posteriori in Section 2.5.

#### Néel magnetization

Substituting Equation (21) into Equation (5) gives

(22) $$N^{2}\left(T,P\right)≃\left\{\begin{array}{cc} 0, & T\geq T_{c},\\ \frac{{\tilde{b}}_{20N}}{{\tilde{b}}_{4N}}\;\left(T_{c}-T\right)+\Delta N_{t}^{2}, & T\leq T_{c}, \end{array}\right.$$

with

(23) $$\Delta N_{t}^{2}=\frac{{\tilde{b}}_{20N}}{{\tilde{b}}_{4N}}\left({\hat{T}}_{N}-T_{c0}\right),\;{\hat{T}}_{N}\equiv {\tilde{T}}_{N}+\frac{{\tilde{g}}_{1N}}{{\tilde{b}}_{20N}},$$

where ${\hat{T}}_{N}$ is the AFM instability temperature in the fully charge‐transferred state (ν=1) at ambient pressure. Pressure enters Equation (22) only through $T_{c}$: the magnetization profile shifts rigidly with the transition line, and the spontaneous magnetization $\Delta N_{t}$ is pressure independent (see Equation (19b)), consistent with experiment [21].

#### Volume

Equation (9) becomes

(24) $$V\left(T,P\right)≃\left\{\begin{array}{cc} 1+\alpha_{\mathrm{HT}}\;\left(T-T_{c0}\right)-\frac{P}{B_{\mathrm{HT}}}, & T\geq T_{c},\\ 1+\alpha_{\mathrm{LT}}\;\left(T-T_{c0}\right)-\frac{P}{B_{\mathrm{LT}}}+\left|\Delta V_{t}\right|, & T\leq T_{c}, \end{array}\right.$$

where we have chosen the undeformed reference state as the HT phase at the ambient‐pressure transition, $T_{0}^{\prime }=T_{c0}$, so that $V(T_{c0}^{+},0)=1$. The coefficients of thermal expansion and the bulk moduli of the two phases are

(25) $$\alpha_{\mathrm{HT}}=\alpha ,\;\alpha_{\mathrm{LT}}=\alpha +\frac{g_{3N}\;{\tilde{b}}_{20N}}{2B\;{\tilde{b}}_{4N}},$$

and

(26) $$B_{\mathrm{HT}}=B,\;\frac{1}{B_{\mathrm{LT}}}=\frac{1}{B}+\frac{g_{3N}^{2}}{2B^{2}\;{\tilde{b}}_{4N}}.$$

In the HT phase the volume is governed solely by the bare thermal expansion and compressibility. In the LT phase, the ICT and the AFM ordering generate the volume discontinuity $\Delta V_{t}$ of Equation (19a) (with $\Delta V_{t}<0$ across the ACFO family), while the magnetoelastic coupling $g_{3N}$ modifies both the thermal expansion and the elastic response. Combining Equations (25) and (26) with Equation (16) shows that the changes in thermal expansivity and compressibility across the phase transition are constrained by the slope of the phase boundary,

(27) $$\alpha_{\mathrm{LT}}-\alpha_{\mathrm{HT}}=\frac{{\tilde{b}}_{20N}^{\;2}}{2\;{\tilde{b}}_{4N}}\;\frac{dT_{c}}{\mathrm{dP}},\;\frac{1}{B_{\mathrm{LT}}}-\frac{1}{B_{\mathrm{HT}}}=\left(\alpha_{\mathrm{LT}}-\alpha_{\mathrm{HT}}\right)\frac{dT_{c}}{\mathrm{dP}}.$$

Since $dT_{c}/dP<0$, the theory predicts $\alpha_{\mathrm{LT}}<\alpha_{\mathrm{HT}}$, as observed [21], together with a softening of the AFM phase, $B_{\mathrm{LT}}<B_{\mathrm{HT}}$.

#### Entropy

Similarly, Equation (10) reduces to

(28) $$\begin{array}{c} S\left(T,P\right)≃\left\{\begin{array}{cc} S_{0}+\alpha_{\mathrm{HT}}^{2}B_{\mathrm{HT}}\;\left(T-T_{c0}\right)-\alpha_{\mathrm{HT}}\;P, & T\geq T_{c},\\ S_{0}+\alpha_{\mathrm{HT}}^{2}B_{\mathrm{HT}}\;\left(T-T_{c0}\right)-\frac{{\left(\alpha_{\mathrm{LT}}-\alpha_{\mathrm{HT}}\right)}^{2}}{B_{\mathrm{LT}}^{-1}-B_{\mathrm{HT}}^{-1}} & \\ \;\left(T_{c0}-T\right)-\Delta S_{t}-\alpha_{\mathrm{LT}}\;P, & T\leq T_{c}, \end{array}\right. \end{array}$$

where we have used Equations (18) and (27) to collect the pressure terms and the identity

(29) $$\frac{{\left(\alpha_{\mathrm{LT}}-\alpha_{\mathrm{HT}}\right)}^{2}}{B_{\mathrm{LT}}^{-1}-B_{\mathrm{HT}}^{-1}}=\frac{{\tilde{b}}_{20N}^{\;2}}{2\;{\tilde{b}}_{4N}},$$

which follows from Equation (27), and where, from Equation (19b) with $\Delta \nu_{t}=-1$,

(30) $$\Delta S_{t}={\tilde{a}}_{10}+\frac{{\tilde{b}}_{20N}}{2}\;\Delta N_{t}^{2}.$$

Equations (24) and (28) are thus expressed entirely in terms of the measurable response functions: $\alpha_{\mathrm{HT}}$, $\alpha_{\mathrm{LT}}$, $B_{\mathrm{HT}}$, and $B_{\mathrm{LT}}$; and the discontinuities $\Delta V_{t}$ and $\Delta S_{t}$. We emphasize their microscopic origin within the step ansatz: since ν does not change within each phase, the charge sector contributes only the discontinuities, through $g_{2}$ and ${\tilde{a}}_{10}$ (Equations (19a) and (30)), whereas the differences in thermal expansion and bulk modulus between the phases arise entirely from the magnetic sector through the magnetoelastic coupling $g_{3N}$ [Equations (25) and (26)]; the left‐hand side of Equation (29) is the mean‐field AFM contribution to the entropy slope below $T_{c}$.

#### Magnetic susceptibility

Equation (11) becomes

(31) $$\mu_{0}\;\chi^{-1}\left(T,P\right)≃\left\{\begin{array}{cc} {\tilde{b}}_{20M}\left[T-\theta_{\mathrm{CW}}\left(P\right)\right], & T\geq T_{c},\\ {\tilde{b}}_{20M}\left[T-\theta_{\mathrm{CW}}\left(P\right)\right]-{\tilde{g}}_{1M} & \\ \;+3\;{\tilde{b}}_{4NM}\;N^{2}\left(T,P\right), & T\leq T_{c}, \end{array}\right.$$

with the pressure‐dependent Curie–Weiss temperature

(32) $$\theta_{\mathrm{CW}}\left(P\right)={\tilde{T}}_{M}-\frac{\lambda }{{\tilde{b}}_{20M}}+\frac{g_{3M}}{B\;{\tilde{b}}_{20M}}\;P.$$

The HT phase exhibits Curie–Weiss behavior, whose ambient‐pressure fit determines $\theta_{\mathrm{CW}}\left(0\right)={\tilde{T}}_{M}-\lambda /{\tilde{b}}_{20M}$. In the LT phase, $\chi^{-1}$ remains linear in T with slope ${\tilde{b}}_{20M}-3\;{\tilde{b}}_{4NM}{\tilde{b}}_{20N}/{\tilde{b}}_{4N}$ and acquires a discontinuity $3\;{\tilde{b}}_{4NM}\Delta N_{t}^{2}-{\tilde{g}}_{1M}$ at the transition.

#### Pressure‐induced entropy changes

Inserting Equations (21) and (22) into Equation (12) yields, for $dT_{c}/dP<0$ and P>0,

(33) $$\Delta S\left(T,0\to P\right)≃\left\{\begin{array}{cc} -\alpha_{\mathrm{HT}}\;P, & T\geq T_{c0},\\ \Delta S_{t}+\frac{{\left(\alpha_{\mathrm{LT}}-\alpha_{\mathrm{HT}}\right)}^{2}}{B_{\mathrm{LT}}^{-1}-B_{\mathrm{HT}}^{-1}}\;\left(T_{c0}-T\right) & \\ \;-\alpha_{\mathrm{HT}}\;P, & T_{c}\leq T\leq T_{c0},\\ -\alpha_{\mathrm{LT}}\;P, & T\leq T_{c}. \end{array}\right.$$

Outside the transition window $T_{c}\leq T\leq T_{c0}$, the response reduces to that of a single phase; in particular, the AFM contribution $\Delta S_{\mathrm{AFM}}$ of Equation (12) does not vanish below $T_{c}$ but combines with the bare term −αP to produce $-\alpha_{\mathrm{LT}}P$. Inside the window, the pressure‐induced LT → HT conversion produces an inverse BC effect of magnitude set by $\Delta S_{t}$, reduced by the thermal expansion of the HT phase and tilted by the AFM entropy slope of Equation (29); at the ambient‐pressure transition temperature, $\Delta S\left(T_{c0},0\to P\right)=\Delta S_{t}-\alpha_{\mathrm{HT}}\;P$. Equation (33) is thus expressed entirely in terms of measurable quantities and makes explicit that the intrinsic thermal expansion of both phases shapes the barocaloric response, a point we quantify for NCFO in Section 3.

### 2.5 Parametrization For NCFO

We now apply the framework to NCFO. From linear fits to the two branches of the unit‐cell volume and from the Néel magnetization and magnetic susceptibility [21], we take

(34) $$\begin{array}{cccc} \nu_{H} & =0, & \nu_{L} & =1,\\ T_{c0} & =310\;K, & \frac{dT_{c}}{dP} & =-36.1\times 10^{-9}\;K\;{\mathrm{Pa}}^{-1},\\ \Delta N_{t} & =3.59\;\mu_{B}/{\mathrm{Fe}}^{3+}, & \Delta V_{t} & =-1.71\times 10^{-2},\\ \alpha_{\mathrm{HT}} & =46.1\times 10^{-6}\;K^{-1}, & \alpha_{\mathrm{LT}} & =18.8\times 10^{-6}\;K^{-1}. \end{array}$$

Since the bulk modulus of NCFO is currently unknown, we set B=118 GPa, equal to that of the HT phase of LCFO [37].

The closed forms of Section 2.4 can be solved directly for the model parameters in terms of the measured inputs in Equation (34). The expansivity $\alpha_{\mathrm{HT}}$ fixes the bare α through Equation (25); the slope $dT_{c}/dP$ fixes the coupling ratios $g_{2}/{\tilde{a}}_{10}=g_{3N}/{\tilde{b}}_{20N}=B\;dT_{c}/dP$ through Eqs. (15)–(17); and the Clausius–Clapeyron relation (20) fixes the transition entropy, $\Delta S_{t}=\Delta V_{t}\;{\left(dT_{c}/dP\right)}^{-1}=473\;\mathrm{kJ}\;m^{-3}\;K^{-1}$. As a consistency check, the latent heat implied by these inputs, $L=T_{c0}\;\Delta S_{t}≃147\;\mathrm{MJ}\;m^{-3}$ agrees within ≃6% with the calorimetric value $157\;\mathrm{MJ}\;m^{-3}$ [21], which was not used in the inversion.

Equation (30) fixes only the sum ${\tilde{a}}_{10}+\left({\tilde{b}}_{20N}/2\right)\Delta N_{t}^{2}=\Delta S_{t}$, so one further input is required to partition the transition entropy between the charge and magnetic channels. We supply it by anchoring the magnetic sector on the intrinsic ordering scale of the charge‐transferred state. An S=5/2 Brillouin fit to the refined ${\mathrm{Fe}}^{3+}$ moment in the low‐temperature phase projects an intrinsic magnetic transition temperature of ≃643 K [21]. Identifying this with the AFM instability temperature of the fully charge‐transferred (ν=1) state, we set ${\hat{T}}_{N}=643$ K. Through Equations (23), (27), and (30) this determines the magnetic sector completely: ${\tilde{b}}_{20N}$ and ${\tilde{b}}_{4N}$ follow from the ratio ${\tilde{b}}_{20N}/{\tilde{b}}_{4N}=\left(N_{0}^{2}-\Delta N_{t}^{2}\right)/T_{c0}$ and the expansivity difference $\alpha_{\mathrm{LT}}-\alpha_{\mathrm{HT}}$, and ${\tilde{a}}_{10}=\Delta S_{t}-\left({\tilde{b}}_{20N}/2\right)\Delta N_{t}^{2}=2.22\times 10^{5}\;J\;m^{-3}\;K^{-1}$, so that the charge and magnetic channels carry comparable fractions (47% and 53%) of the transition entropy. The couplings $g_{2}$ and $g_{3N}$ then follow from the ratios fixed by $dT_{c}/dP$, and stationarity of the HT minimum at $\nu_{H}=0$ at the ambient‐pressure transition, Equation (14a), sets ${\tilde{T}}_{\nu }=T_{c0}$.

With these values the mean‐field profile of Equation (22) extrapolates to $N_{0}=4.99\;\mu_{B}/{\mathrm{Fe}}^{3+}$ at T=0K, in close agreement with the full S=5/2 moment of $5\;\mu_{B}$ expected for ${\mathrm{Fe}}^{3+}$. This value is implied by the anchor rather than fitted independently: ${\hat{T}}_{N}$, $\Delta N_{t}$, and $T_{c0}$ together fix $N_{0}$ through Equation (23). The moment measured at 17 K, $3.97\;\mu_{B}$ [21], is somewhat smaller, as discussed at the end of this section.

At $\left(T_{c0},\;P=0\right)$ with $\nu_{H}=0$ and $\nu_{L}=1$, the coexistence and stationarity conditions in Equations (13) and (14b) are linear in ${\tilde{a}}_{2}$ and $a_{3}$ and are solved for them exactly. The remaining coefficients $a_{4}$, $a_{5}$, and $a_{6}$ are constrained only by boundedness ($a_{6}>0$) and by the requirement that the equilibrium ν(T) reproduce the step profile of Equation (21). This requirement does not determine these parameters uniquely: a wide family of $\left(a_{4},a_{5},a_{6}\right)$ reproduces the profile equally well, so there is no single data‐driven value for these coefficients. We therefore fix them to a representative set. No measured quantity depends on this choice: the relative volume, entropy, staggered magnetization, susceptibility, and the phase boundary (Equations (24)–(33), (31), and (18)) are determined by the positions of the free‐energy minima and by the transition conditions at $T_{c0}$, not by the shape of $\hat{G}\left(\nu \right)$ between them.

The magnetic parameters ${\tilde{b}}_{20M}$, $\theta_{\mathrm{CW}}\left(0\right)$, ${\tilde{b}}_{4NM}$, and ${\tilde{g}}_{1M}$ are obtained from the susceptibility in three steps. The Curie–Weiss regime of the HT phase ($T≳T_{c0}+20$ K) fixes ${\tilde{b}}_{20M}$ and $\theta_{\mathrm{CW}}\left(0\right)$ through Equation (32); the slope of $\chi^{-1}\left(T\right)$ in the LT phase, ${\tilde{b}}_{20M}-3{\tilde{b}}_{4NM}{\tilde{b}}_{20N}/{\tilde{b}}_{4N}$, fixes ${\tilde{b}}_{4NM}$; and the discontinuity of $\chi^{-1}$ at $T_{c0}$, $3{\tilde{b}}_{4NM}\Delta N_{t}^{2}-{\tilde{g}}_{1M}$, fixes ${\tilde{g}}_{1M}$. In the absence of pressure‐dependent susceptibility data, $g_{3M}$, and hence ${\tilde{b}}_{4M}$, remain undetermined. Table 1 lists the resulting values.

**TABLE 1:** Landau parameter values for NCFO. The AFM instability temperature of the fully charge‐transferred state, ${\hat{T}}_{N}={\tilde{T}}_{N}+{\tilde{g}}_{1N}/{\tilde{b}}_{20N}=643$ K, is anchored on the projected ordering temperature [21]; $\theta_{\mathrm{CW}}\left(0\right)={\tilde{T}}_{M}-\lambda /{\tilde{b}}_{20M}=230$ K is obtained from the Curie–Weiss fit. ${\tilde{T}}_{M}$ and λ enter only through this combination. The coefficients $a_{4}$, $a_{5}$, $a_{6}$ are representative values (see text). We found no data to fit $g_{3M}$, so it and ${\tilde{b}}_{4M}$ are undetermined, but avoidance of a FM ground state requires ${\tilde{b}}_{4M}≳1.3\times 10^{-15}$ J m $A^{-4}$.

Parameter	Value	Units
${\tilde{a}}_{10}$	$2.22\times 10^{5}$	J $m^{-3}$ $K^{-1}$
${\tilde{a}}_{2}$	$1.75583\times 10^{10}$	J $m^{-3}$
$a_{3}$	$-4.76120\times 10^{10}$	J $m^{-3}$
$a_{4}$	$2.20\times 10^{10}$	J $m^{-3}$
$a_{5}$	$8.00\times 10^{9}$	J $m^{-3}$
$a_{6}$	$2.00\times 10^{8}$	J $m^{-3}$
${\tilde{b}}_{20N}$	$1.1539\times 10^{-6}$	J $m^{-1}$ $A^{-2}$ $K^{-1}$
${\tilde{b}}_{4N}$	$8.8229\times 10^{-16}$	J m $A^{-4}$
${\tilde{g}}_{1N}$	$6.7205\times 10^{-4}$	J $m^{-1}$ $A^{-2}$
$g_{2}$	$-9.4568\times 10^{8}$	J $m^{-3}$
$g_{3N}$	$-4.9154\times 10^{-3}$	J $m^{-1}$ $A^{-2}$
${\tilde{T}}_{\nu }$	310	K
${\tilde{T}}_{N}$	60.6	K
α	$46.1\times 10^{-6}$	$K^{-1}$
B	$118\times 10^{9}$	Pa
${\tilde{b}}_{20M}$	$4.30\times 10^{-6}$	J $m^{-1}$ $A^{-2}$ $K^{-1}$
${\tilde{b}}_{4NM}$	$4.01\times 10^{-16}$	J m $A^{-4}$
${\tilde{g}}_{1M}$	$-9.09\times 10^{-4}$	J $m^{-1}$ $A^{-2}$

We verify the construction a posteriori. With the parameters of Table 1, the equilibrium condition given in Equation (8) reproduces the step profile of Equation (21) to within 1% over 0K≤T≤400K, with all solutions corresponding to absolute minima of $\hat{G}$. The same equilibria are recovered by direct minimization of the full effective free energy $\tilde{G}\left(\nu ,N,M\right)$ of Equation (3) over all three OPs, with M=0 in the equilibrium AFM phase and $\mu_{0}\chi^{-1}>0$ throughout; the PM branch develops a FM instability below $\theta_{\mathrm{CW}}\left(0\right)$ that remains metastable relative to the charge‐transferred ground state for ${\tilde{b}}_{4M}≳1.3\times 10^{-15}\;J\;m\;A^{-4}$. The choice of ${\tilde{a}}_{10}$ does not affect the thermodynamic observables of Equations (24)–(33), which depend on it only through $\Delta S_{t}$ and the renormalized expansivity and compressibility fixed by Equation (34); it enters the charge potential through ${\hat{a}}_{1}$ and ${\hat{a}}_{2}$ (Equation (7)), which is absorbed in the re‐solved ${\tilde{a}}_{2}$ and $a_{3}$.

Finally, the linear mean‐field profile of Equation (22) describes the Néel data within $≃0.05\;\mu_{B}$ (rms) over $T_{c0}-120\;K≲T\leq T_{c0}$, but its extrapolated $N_{0}=4.99\;\mu_{B}$ exceeds the moment measured at 17 K, $3.97\;\mu_{B}$, because N(T) saturates at low temperature whereas the linear profile does not. This is a generic limitation of the linear‐in‐temperature coefficients: the third law of thermodynamics requires ∂S/∂T→0 as T→0, whereas Eqs. (10) and (22) give a nonvanishing entropy slope there. As the present work concerns the strongly first‐order regime near $T_{c0}$, this low‐temperature discrepancy does not affect our results, and we leave a saturating description of the coefficients to future work.

## 3 Results and Discussion

**FIGURE 2:**
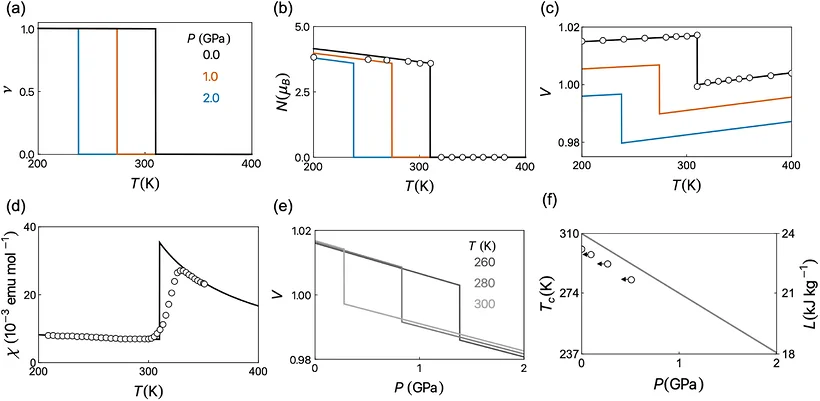
Calculated thermodynamic properties of NCFO across the intersite‐charge‐transfer transition. Temperature dependence of (a) the charge‐transfer order parameter, (b) the staggered magnetization per ${\mathrm{Fe}}^{3+}$, (c) the relative volume, and (d) the molar magnetic susceptibility. (e) Pressure dependence of the relative volume along selected isotherms. (f) Temperature–pressure phase boundary, with the transition temperature $T_{c}$ (left axis) and latent heat $L=T_{c}\;\Delta S_{t}$ (right axis); the open circles correspond to the left axis. The color scheme in (b), (c), and (d) follows the same pressure assignments as the legend in (a). Experimental data (open circles) taken from Ref. [21].

With the NCFO parameters of Table 1, we solve the stationarity condition in Equation (8) for the equilibrium charge‐transfer OP ν(T,P) and evaluate the relative volume, entropy, staggered magnetization, and susceptibility given by Equations (9)–(11). Figure 2 compiles these results: panels (a)–(d) show the temperature dependence at P=0, 1, and 2 GPa; panel (e) the relative volume along three isotherms as a function of pressure; and panel (f) the transition temperature and latent heat along the phase boundary. Open circles are the NCFO measurements of Ref. [21]; the solid curves are computed from the inputs of Equation (34).

The charge‐transfer OP (Figure 2a) switches between ν≃0 and ν≃1 at $T_{c}\left(P\right)$, the equilibrium profile reproducing the step ansatz of Equation (21) (Section 2.5), with the transition moving from $T_{c0}=310$ K to 274 and 238 K at 1 and 2 GPa. The staggered magnetization (Figure 2b) appears discontinuously at $T_{c}$ and grows on further cooling. The linear mean‐field profile of Equation (22) follows the refined B‐site Fe moment of Ref. [21] to within $0.05\;\mu_{B}$ (rms) over $T_{c0}-120\;K≲T\leq T_{c0}$, the transition jump $\Delta N_{t}=3.59\;\mu_{B}$ matching the abrupt, first‐order onset of the moment seen in the neutron data. Below this range the linear profile exceeds the measured low‐temperature moment, a limitation of the linear‐in‐T coefficients discussed in Section 2.5 that does not affect the strongly first‐order regime of interest here.

The relative volume (Figure 2c) consists of two linear branches separated by the volume jump $\Delta V_{t}$, with the low‐temperature phase larger by $|\Delta V_{t}|≃1.7\%$ (negative‐thermal‐expansion‐like expansion on cooling) and distinct expansivities, $\alpha_{\mathrm{HT}}=46.1\times 10^{-6}\;K^{-1}>\alpha_{\mathrm{LT}}=18.8\times 10^{-6}\;K^{-1}$. The ambient‐pressure branch reproduces the synchrotron X‐ray diffraction volume [21] through the transition; under pressure both branches shift downward through compression and the step tracks the phase boundary to lower temperature.

The magnetic susceptibility (Figure 2d) follows Curie–Weiss behavior in the PM phase, with $\theta_{\mathrm{CW}}\left(0\right)=230$ K, and is suppressed and weakly temperature dependent in the AFM phase, with a discontinuity at $T_{c}$. The model produces a sharp cusp at $T_{c}$, whereas the experimental data show a rounded maximum slightly above it, reflecting the finite width of the real transition. In the absence of pressure‐dependent susceptibility data, this panel is shown at ambient pressure only.

Panel (e) is a prediction. At fixed temperature the volume decreases linearly within each phase, with slopes $-1/B_{\mathrm{LT}}$ at low pressure and $-1/B_{\mathrm{HT}}$ at high pressure, and drops by $≃|\Delta V_{t}|$ at the transition pressure $P_{c}\left(T\right)=\left(T_{c0}-T\right)/\left|dT_{c}/dP\right|$, equal to 0.28, 0.83, and 1.38 GPa at 300, 280, and 260 K. To our knowledge, there are no published measurements of V(P) across the transition for NCFO. The predicted linear compression within each isostructural phase, together with a downward step of order 1–2%, reproduces the pressure‐driven intersite charge transfer measured in the sister compound LCFO [37], in which hydrostatic pressure converts the ambient, charge‐transferred ${\mathrm{Cu}}^{3+}$/${\mathrm{Fe}}^{3+}$ phase (the analogue of the LT phase here) to the high‐pressure ${\mathrm{Cu}}^{2+}$/${\mathrm{Fe}}^{3.75+}$ phase (the analogue of the HT phase) through a ≃1.7% isostructural volume contraction. The sub‐GPa transition pressures predicted for NCFO lie well below the ≃3 GPa of LCFO, tracking the lower ambient transition temperature of NCFO (310 K vs. 393 K).

The model and the LCFO data disagree, however, on the ordering of the two compressibilities. Equation (26) gives $B_{\mathrm{LT}}^{-1}-B_{\mathrm{HT}}^{-1}=g_{3N}^{2}/\left(2B^{2}{\tilde{b}}_{4N}\right)>0$, so the model predicts the charge‐transferred AFM phase to be the softer of the two, $B_{\mathrm{LT}}<B_{\mathrm{HT}}$. The LCFO measurements [37] show the opposite: a bulk modulus of 133GPa for the charge‐transferred LT phase against 118GPa for the HT phase. Given the agreement with the NCFO data above and the close empirical similarity across the ACFO family [10], we attribute this discrepancy to the model rather than to a genuine difference between NCFO and LCFO. In our model, volume strain couples linearly to the charge and magnetizations, which yields a jump $B_{\mathrm{LT}}^{-1}-B_{\mathrm{HT}}^{-1}$ that is sign‐definite and the AFM phase is necessarily the softer one, for any choice of parameters. The same magnetoelastic coupling $g_{3N}$ that yields the observed expansivity ordering $\alpha_{\mathrm{LT}}<\alpha_{\mathrm{HT}}$ thus fixes the compressibility ordering as well, the two being tied through Equation (27); retuning the parameters cannot reverse one without losing the other. Capturing $B_{\mathrm{LT}}>B_{\mathrm{HT}}$ requires strain couplings beyond linear order, e.g., a biquadratic term $\eta^{2}\nu$ or $\eta^{2}N^{2}$ that renormalizes the modulus differently in the two phases and can stiffen the charge‐transferred phase. We leave such an extension to future work, noting that a pressure‐resolved V(P) measurement across the transition in NCFO would test both the predicted morphology of panel (e) and the compressibility ordering directly.

The transition temperature (Figure 2f, left axis) is linear in pressure, Equation (18), as required by the concomitance constraint in Equation (17). Its slope, $dT_{c}/dP=-36.1\;K\;{\text{GPa}}^{-1}$, is the value measured in Ref. [21] and used as an input in the parametrization (Section 2.5); the open circles are the cooling‐run transition temperatures of that work, which lie on a line of the same slope offset ≃10 K below the computed boundary, the offset reflecting the thermal hysteresis of the first‐order transition. Because $\Delta S_{t}$ is pressure independent [Equation (19b)], the latent heat $L=T_{c}\;\Delta S_{t}$ is likewise linear in pressure (Figure 2f, right axis), decreasing from $23.5\;\text{kJ}\;{\text{kg}}^{-1}$ at ambient pressure to $18\;\text{kJ}\;{\text{kg}}^{-1}$ at 2 GPa; the ambient‐pressure value agrees with calorimetry to within 6% (Section 2.5).

The framework also rationalizes the thermal‐energy‐storage behavior of the ACFO family [22]. In our model, the transition entropy (Equation (19b)) is determined entirely by the OP discontinuities and the coefficients ${\tilde{a}}_{10}$, ${\tilde{b}}_{20N}$ of the charge and magnetic sectors, which are intrinsic properties of the Cu–Fe ICT transition. To the extent that A‐site substitution tunes $T_{c}$ without altering this electronic transition, $\Delta S_{t}$ is preserved while the latent heat $L=T_{c}\;\Delta S_{t}$ scales with $T_{c}$. Indeed, this is what is observed: across A = La, Pr, Nd, Sm, Eu, and Gd the transition entropy remains nearly constant (≃8% reduction per 100 K) while the latent heat falls in proportion to the transition temperature [22].

**FIGURE 3:**
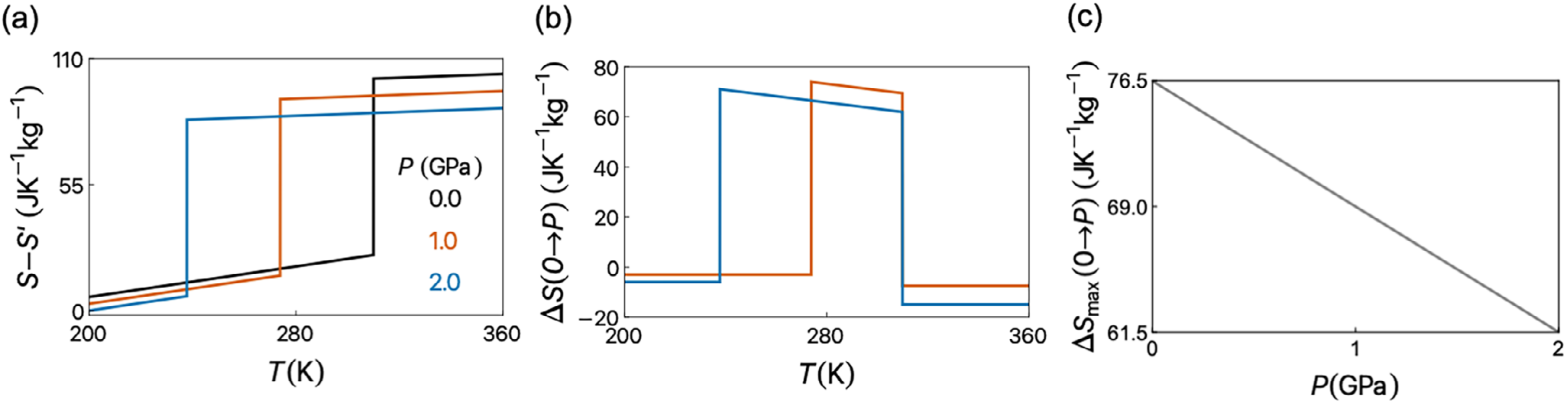
Calculated barocaloric response of NCFO. (a) Temperature dependence of the entropy at P=0, 1, and 2 GPa, plotted relative to a common reference $S^{\prime }=S(T=280\;K,\;P=2\;\text{GPa})$. (b) Isothermal entropy change upon compression, ΔS(0→P). The color scheme follows the same pressure assignments as the legend in (a). (c) Maximum isothermal entropy change upon compression, $\Delta S_{\max}\left(0\to P\right)$, vs. pressure.

We now turn to the BC response, obtained from Equations (28) and (33). Figure 3a shows the entropy S(T) at P=0, 1, and 2 GPa, plotted relative to a common reference $S^{\prime }=S(T=280\;K,P=2\;\mathrm{GPa})$. Within each phase S rises linearly with temperature, with slopes set by the single‐phase expansivities and moduli, and at $T_{c}\left(P\right)$ it jumps by the transition entropy $\Delta S_{t}≃76.5\;J\;K^{-1}{\text{kg}}^{-1}$ (obtained from Equation (20)) rather than from calorimetry, and ≃9% below the calorimetric value $84.2\;J\;K^{-1}{\text{kg}}^{-1}$ [21]), the high‐temperature phase carrying the larger entropy. Increasing pressure shifts the curve rigidly to lower temperature along the phase boundary and downward through the single‐phase $-\alpha_{\mathrm{HT}}P$ and $-\alpha_{\mathrm{LT}}P$ terms.

The isothermal entropy change upon compression, ΔS(0→P) (Figure 3b, Equation (33)), is positive across the transition window: an inverse BC effect. The inverse sign is a direct consequence of $dT_{c}/dP<0$, compression converting the lower‐entropy charge‐transferred phase into the higher‐entropy high‐temperature phase, and is the hydrostatic counterpart of the Clausius–Clapeyron relation $dT_{c}/dP=\Delta V_{t}/\Delta S_{t}<0$ of Section 2.3.2, with $\Delta V_{t}<0$ and $\Delta S_{t}>0$. Outside the window the response reduces to the single‐phase values $-\alpha_{\mathrm{HT}}P$ above $T_{c0}$ and $-\alpha_{\mathrm{LT}}P$ below $T_{c}$, the conventional (co‐aligned) contributions of each phase.

The peak of the response, $\Delta S_{\max}\left(0\to P\right)=\Delta S\left(T_{c0},0\to P\right)=\Delta S_{t}-\alpha_{\mathrm{HT}}P$, decreases linearly with pressure (Figure 3c), from $\Delta S_{t}$ at ambient pressure with a slope fixed by the high‐temperature‐phase expansivity $\alpha_{\mathrm{HT}}$. The intrinsic thermal expansion of the high‐temperature phase thus sets the rate at which the inverse effect diminishes under pressure.

These results may be compared with the BC effects determined by the indirect method [21]. Both the model and the indirect method yield an inverse BC effect, as anticipated from $dT_{c}/dP<0$, and of comparable magnitude: the indirectly determined $\Delta S_{\max}$ reaches $65.1\;J\;K^{-1}{\text{kg}}^{-1}$ at ≃0.5 GPa, 77% of the transition entropy. The pressure dependence, however, differs in sign: the model predicts $\Delta S_{\max}$ to decrease with pressure (Equation (33)), whereas the indirectly determined value increases.

The model decrease originates in the single‐phase $-\alpha_{\mathrm{HT}}P$ term, which the indirect method [21] omits by referencing each isobar to a common high‐temperature value; this accounts for the differing trend relative to a thermal‐expansion‐free reconstruction. The pronounced increase of the indirectly determined $\Delta S_{\max}$ toward $\Delta S_{t}$ with pressure, by contrast, reflects the finite width of the real first‐order transition: at low pressure the transition windows at P and at ambient pressure overlap, so only part of $\Delta S_{t}$ is swept reversibly, and $\Delta S_{\max}$ approaches $\Delta S_{t}$ only as pressure separates them. A mean‐field Landau theory describes a first‐order transition as a discontinuous jump of the equilibrium OP at $T_{c}$, with no coexistence width; it therefore delivers the full $\Delta S_{t}$ at infinitesimal pressure separation and cannot reproduce this growth, which lies beyond the homogeneous, mean‐field regime the present model targets.

## 4 Conclusions

We have developed a minimal Landau theory of the concomitant intersite charge‐transfer, antiferromagnetic, and isostructural phase transitions in the ${\mathrm{ACu}}_{3}{\mathrm{Fe}}_{4}O_{12}$ quadruple perovskites with A = La, Pr, Nd, Sm, Eu, Gd, Tb. Taking the charge‐transfer order parameter as primary and enslaving the staggered magnetization and the volume strain to it yields an effective free energy in which the volume and entropy discontinuities, the phase‐specific thermal expansivities, the bulk moduli, and the phase‐boundary slope are expressed in closed form and tied to one another through the model couplings. A step ansatz, justified by the abrupt charge transfer seen across the family, reduces these to explicit functions of a small set of measurable inputs and permits a direct inversion of the data.

Applied to ${\mathrm{NdCu}}_{3}{\mathrm{Fe}}_{4}O_{12}$, the theory reproduces the charge‐transfer, staggered‐magnetization, susceptibility, and volume responses through the transition from parameters fixed by independent structural, magnetic, and calorimetric data. The latent heat obtained from the Clausius–Clapeyron equation agrees with calorimetry to within 6%, and the partition of the transition entropy into comparable charge and magnetic contributions follows from anchoring the magnetic sector on the intrinsic ordering scale of the charge‐transferred state. Because the transition entropy is pressure independent, the latent heat scales with the transition temperature, which accounts for its near‐constancy under A‐site substitution and its proportional decline with the substitution‐tuned $T_{c}$. The negative phase‐boundary slope produces an inverse barocaloric effect, comparable in magnitude to the quasi‐direct determination, with the high‐temperature expansivity setting the rate at which the effect decreases with pressure.

The closed‐form relations also indicate how the barocaloric response might be tuned. The pressure required to drive the transition at an operating temperature T is $P_{c}\left(T\right)=\left(T_{c0}-T\right)/\left|dT_{c}/dP\right|$, so the driving pressure can be reduced by placing the ambient transition temperature $T_{c0}$ near the operating window through A‐site substitution and by increasing the magnitude of the phase‐boundary slope. From the Clausius–Clapeyron relation $dT_{c}/dP=\Delta V_{t}/\Delta S_{t}$, the latter requires a large volume collapse relative to the transition entropy. The transition entropy, $\Delta S_{t}=-{\tilde{a}}_{10}\;\Delta \nu_{t}+\left({\tilde{b}}_{20N}/2\right)\;\Delta N_{t}^{2}$, can in turn be enhanced through either the charge channel or magnetic channel. The charge contribution is favored by a large ${\tilde{a}}_{10}$ and by a large charge transfer at the transition (in ACFO, $|\Delta \nu_{t}|=1$, already the maximum).  The magnetic contribution grows with the square of the ordered moment and is therefore favored by a high‐spin B‐site cation. These design objectives are partly competing: increasing $\Delta S_{t}$ at fixed $\Delta V_{t}$ reduces $|dT_{c}/\mathrm{dP}|$ and therefore increases the required driving pressure. An advantageous material must therefore combine a large transition entropy with a comparably large isostructural volume change, so that $|\Delta V_{t}|/\Delta S_{t}$, and hence $|dT_{c}/dP|$, remains large. Within the present minimal description, ${\mathrm{ACu}}_{3}{\mathrm{Fe}}_{4}O_{12}$ comes close to this combination: its phase transition brings together a complete intersite charge transfer, a large ${\mathrm{Fe}}^{3+}$ moment, and a ≃2% volume collapse.

Our model has two clear limitations. The more consequential is that it predicts the charge‐transferred phase to be the more compressible of the two, $B_{\mathrm{LT}}<B_{\mathrm{HT}}$, whereas the measured bulk moduli of ${\mathrm{LaCu}}_{3}{\mathrm{Fe}}_{4}O_{12}$ run the other way. This follows unavoidably from coupling the strain to the charge and magnetic sectors only linearly, which renders the compressibility jump sign‐definite; reversing it to $B_{\mathrm{LT}}>B_{\mathrm{HT}}$ calls for strain couplings beyond linear order, which we leave to future work. The second is intrinsic to the mean‐field description: a homogeneous Landau theory renders the transition a discontinuous jump of zero coexistence width, and so cannot capture the growth of the indirectly determined isothermal entropy change toward the full transition entropy as pressure draws the transition clear of its ambient‐pressure position.

Two measurements would sharpen the picture. A pressure‐resolved determination of the volume across the transition in ${\mathrm{NdCu}}_{3}{\mathrm{Fe}}_{4}O_{12}$ would test the predicted compression morphology and the compressibility ordering at once, and pressure‐dependent susceptibility data would fix the magnetoelastic coupling left undetermined here. More broadly, the framework could be adapted to other intersite charge‐transfer [38] and charge‐disproportionation [10] systems showing concomitant first‐order electronic, magnetic, and structural transitions.

## Funding

This work was supported by the Vicerrectoría de Investigación, Universidad de Costa Rica (C5601 and C6208).

## Conflicts of Interest

The authors declares no conflicts of interest.

## Data Availability

The data that support the findings of this study are available from the corresponding author upon reasonable request.
